# New products

**DOI:** 10.1155/S1463924602000226

**Published:** 2002


Journal of Automated Methods & Management in Chemistry
Vol. 24, No. 6 (November–December 2002) pp. 211–212

Taylor & Francis

Taylor & Francis Group

### New products

### Vol. 1B Version 2 Infu-Disk™ Infusion Pumps for Biomedical Research

A new technology ideal for an accurate, efficient fluid delivery devices has been made with a new patent issued to Med-e-Cell which allows for the development of breakthrough fluid delivery devices for healthcare and research. Med-e-Cell's Prime-Mover® technology portfolio was recently strengthened with the addition of a fluid delivery technology that allows for smaller, more flexible device design while increasing efficiency by four times. The technology has been demonstrated in prototypes delivering highly viscous fluids. Tests using these prototypes prove a marked reduction in size and power requirements and show fourfold increase in flow rate range. Med-e-Cell believes this technology, in addition to applications in research and animal health, has far reaching implications for human healthcare. The technology is ideal for ambulatory, low-cost, disposable, infusion devices, including ultra-miniature devices that are not presently available. This technology is capable of delivery accuracies that are only possible with higher cost electromechanical technologies while maintaining a manufactured cost comparable to elastomeric pumps.

*For more information, contact Med-e-Cell. Tel: + 1 858 552 0436; Internet: http://www.medecell.com*

### Med-e-Cell's adds Infu-Disk® sales representation in Florida and Puerto Rico

D&D Medical Sales is now offering Infu-Disk® infusion pumps. Infu-Disk® pumps are now being offered through D&D Medical Sales.

*For more information, contact Infu-Disk®* Tel: + 1 800 472 1203/858 552 0436.

### GENEQ, Inc., adds Infu-Disk® infusion pumps to their product line

GENEQ, Inc., a leading supplier of instruments and equipment for biological research in Canada, adds Infu-Disk® infusion pumps to their product line. Infu-Disk® infusion pumps can be found in their 2002–2003 'Biological Research' catalogue on p. 44.

*For more information or to obtain a catalogue, Tel: + 1 800 463 4363. Internet: http://www.geneq.com or call*

### GENEQ, Kent Scientific and Thomas Scientific

GENEQ joins Kent Scientific (http://www.kentscientific.com) and Thomas Scientific (http://www.thomassci.com) in offering Infu-Disk® infusion products to researchers in North America. Kent Scientific also attended the Experimental Biology Conference in New Orleans, 20–24 April 2002 where it displayed the Infu-Disk® In addition to offering Infu-Disk® infusion pumps and the Infu-Saddle, Kent Scientific offers a complete line of research products including animal anaesthesia equipment, blood pressure monitors, physiological transducers and surgical instruments.

Infu-Disk® pumps and the Infu-Saddle, an ideal solution for quickly and easily securing Infu-Disk® pumps, can be ordered by calling + 1 888 5RATTUS (+ 1 888 572 8887). Internet: http://www.kentscientific.com

### 5 ml Infu-Disk®

- $41.00 standard/$44.00 non-standard.
- Delivery rates: 0.03–1 ml $h^{-1}$.
- Accuracy better than ±10%.
- Available sterile or non-sterile.
- Connector: male Luer lock or cannula Accurate, continuous fluid delivery from a 5-ml reservoir at rates from 0.03 to 1.0 ml $h^{-1}$.
- View performance specification data.
- Exceptionally easy to use and fill.
- View use instructions.

### 10 ml Infu-Disk®

- $41.00 standard/$44.00 non-standard.
- Delivery rates: 0.03–0.5 ml $h^{-1}$.
- Accuracy better than ±10%.
- Available sterile or non-sterile.
- Connector: male Luer lock or cannula Accurate, continuous fluid delivery from a 10-ml reservoir at rates from 0.03 to 0.5 ml $h^{-1}$.
- View performance specification data.
- Exceptionally easy to use and fill.
- View use instructions.

### 10 ml Infu-Disk® HF

- $44.00.
- Delivery rates: 0.5–4.0 ml $h^{-1}$.
- Accuracy better than ±10%.
- Available sterile or non-sterile.
- Connector: male Luer lock or cannula or cannula Accurate, continuous fluid delivery from a 10-ml reservoir at rates from 0.5 and 4.0 ml $h^{-1}$.
- View performance specification data.
- Exceptionally easy to use and fill.
- View use instructions.

All Med-e-Cell products including Infu-Disk® are based on its patented Prime Mover® technology. Prime Mover® is an efficient, low-power, cost-effective means

*Journal of Automated Methods & Management in Chemistry* ISSN 1463–9246 print/ISSN 1464–5068 online © 2002 Taylor & Francis Ltd
http://www.tandf.co.uk/journals
DOI: 10.1080/1463924021000033282

to move fluids, move and generate gases, actuate devices, generate power, etc.

*For further information, contact Med-e-Cell. Tel: + 1 858 552 0781. Internet: http://www.medecell.com*

**More productive chemistry**

RDT's Carousel 6 Place Reaction Station employs traditional format 250-ml flasks for efficient parallel synthesis. The new Carousel 6 Place Reaction Station is a simple, convenient way for chemists to increase the productivity of their chemistry for larger scale synthesis, including process development and the synthesis of intermediates and building blocks. Developed in collaboration with working chemists, this practical yet innovative synthesizer combines the proven technology of the popular 12-place Carousel with familiar 25-ml round-bottomed flasks. Using the same standard hotplate stirrer, PTFE caps and fuzzy logic temperature control, the Carousel 6 Place provides six heated and stirred positions. The 6 Place unit accommodates six 250-ml round bottomed reaction flasks, each with a working volume of 150-ml and a typical reaction scale of 10 g. Its patented design ensures efficient heat transfer and refluxing, enabling it to boil six 150-ml flasks of water in less than 25 min, with new elliptical rare earth stirring bars providing powerful stirring and a deep vortex.

Operating temperature ranges from ambient to 160°C, with accurate temperature control to +/ − 0.5°C. An integral PTFE guard-ring helps protect the user from accidental contact with the heated base.

One Carousel 6 Place unit, which takes up very little fume cupboard space, replaces the work of six individual heating mantles or hotplate stirrers with round-bottomed flask and condenser assemblies, making it a truly cost-effective addition to any chemistry laboratory.

Every aspect of the Carousel 6 Place has been designed for convenience: *the station allows each flask to be clearly visible and easily accessible for addition of reagents and solvents. clip-in aluminium inserts enable the flasks to be quickly inserted and removed and give excellent reflux cooling. And, the traditional format 250-ml reaction flasks are designed to be transferred directly to a standard rotary evaporator*. Like the popular 12 position Carousel, the new 6 Place unit uses the same standard gas-tight PTFE caps for operation under an inert atmosphere and for the addition of reagents via a Suba-seal septa.

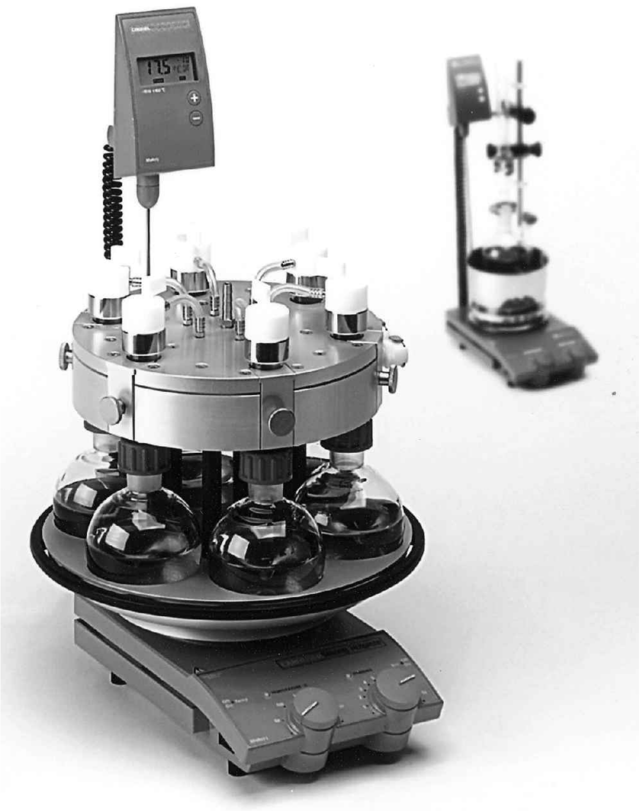

The Carousel 6 Place Unit is completely maintenance free, with no moving parts requiring repairs or servicing. It is easy to set-up with minimal training time.

*For more information, contact Clare Schneider, Marketing Coordinator, Radleys Discovery Technologies Ltd, Shire Hill, Saffron Walden CB11 3AZ, UK. Tel: +44 (0)1799 513320; Fax: +44 (0)1799 513283; e-mail: sales@radleys.co.uk. Internet: http//www.radleys.com*

